# PTHG2 Reduces Bone Loss in Ovariectomized Mice by Directing Bone Marrow Mesenchymal Stem Cell Fate

**DOI:** 10.1155/2021/8546739

**Published:** 2021-11-19

**Authors:** Jiao Chen, Hao Zhang, Xianmin Wu, Fuxiao Wang, Yili Wang, Qianmin Gao, Han Liu, Yan Hu, Jiacan Su, Yingying Jing

**Affiliations:** ^1^Institute of Translational Medicine, Shanghai University, Shanghai 200444, China; ^2^Department of Orthopedics Trauma, Shanghai Changhai Hospital, Naval Medical University, Shanghai 200433, China; ^3^Department of Orthopedics Trauma, Shanghai Zhongye Hospital, Shanghai 201900, China; ^4^Shanghai Clinical Research Center for Aging and Medicine, Shanghai 200040, China

## Abstract

Teriparatide, also known as 1-34 parathyroid hormone (PTH (1-34)), is commonly used for the treatment of osteoporosis in postmenopausal women. But its therapeutic application is restricted by poor metabolic stability, low bioavailability, and rapid clearance. Herein, PTHG2, a glycosylated teriparatide derivative, is designed and synthesized to improve PTH stability and exert more potent antiosteoporosis effect. Surface plasmon resonance (SPR) analysis shows that PTHG2 combines to PTH 1 receptor. Additional acetylglucosamine covalent bonding in the first serine at the N terminal of PTH (1-34) improves stability and increases protein hydrolysis resistance. Intermittent administration of PTHG2 preserves bone quality in ovariectomy- (OVX-) induced osteoporosis mice model, along with increased osteoblastic differentiation and bone formation, and reduced marrow adipogenesis. In vitro, PTHG2 inhibits adipogenic differentiation and promotes osteoblastic differentiation of bone marrow mesenchymal stem cells (BMSCs). For molecular mechanism, PTHG2 directs BMSCs fate through stimulating the cAMP-PKA signaling pathway. Blocking PKA abrogates the pro-osteogenic effect of PTHG2. In conclusion, our study reveals that PTHG2 can accelerate osteogenic differentiation of BMSCs and inhibit adipogenic differentiation of BMSCs and show a better protective effect than PTH (1-34) in the treatment of osteoporosis.

## 1. Introduction

Osteoporosis is a primary public health problem listed by the World Health Organization (WHO) [1]. Recently, imbalance of BMSC osteogenic and adipogenic differentiation in both age-related or postmenopausal osteoporosis has been regarded as a major risk factor [2–4]. Current clinical treatment of osteoporosis is aimed at recovering a normal bone metabolic balance [5, 6].

BMSCs are derived from bone marrow, self-renewal, and multipotential progenitor cells of osteoblasts and adipocytes in appropriate conditions [7, 8]. The balance of adipo-osteogenic differentiation is under a precise control of both biological and mechanical factors. Under aging or pathological conditions like osteoporosis, BMSCs experience differentiation shift from osteoblasts to adipocytes which leads to adipocytes accumulation, aberrant skeletal architecture, and elevated fracture risk [9–11].

Excessive adipogenesis of BMSCs is correlated with osteoporosis progression. Adipose tissue accumulation is commonly observed in osteoporosis, at the expense of impaired osteogenic regeneration and bone formation [12]. The differentiation of BMSCs into adipocytes takes precedence over osteoblasts in osteoporosis [13]. BMSCs differentiate into adipocytes, which competes with bone marrow osteoblast formation [13, 14]. Adipocytes secrete adiponectin which inhibits the osteogenic differentiation of BMSCs [15]. Mir-188 is overexpressed in aged BMSCs, which accelerates adipogenesis and inhibits osteogenesis [16]. Inhibition of Mir-188 reduces adipogenesis and alleviates osteoporosis.

Directing BMSC differentiation fate remains a promising therapeutic tactics in the healing of osteoporosis. Peroxisome proliferator-activated receptors ɣ (PPARɣ) are fat-specific nuclear transcription factors which adjust the expression of related genes in adipocytes. PPARɣ can promote adipocyte propagation and differentiation [17, 18]. Several researches use antagonist drugs of PPARɣ to inhibit adipocyte generation, such as bisphenol-A-diglyceryl ether (BADGE), SR10171, and Oleuropein; all of which have been proved to inhibit the expression of PPARɣ in BMSCs, inhibit adipocyte generation, and enhance BMSCs osteogenic differentiation, thus relieving osteoporosis [14, 19].

Parathyroid hormone and PTH (1-34) as a sort of anabolic therapeutic agent of osteoporosis are widely applied. PTH (1-34) orchestrates multiple cells and signaling pathways that regulate BMSC differentiation. Intermittent administration of PTH (1-34) stimulates bone remodeling and increases bone synthesis [20–24]. PTH (1-34) administration increases nestin^+^ BMSCs *in vivo* [25, 26] and directs BMSC differentiation [27, 28]. However, as a linear polypeptide, PTH (1-34) has inherent disadvantages such as poor metabolic stability, low bioavailability, and rapid clearance [29]. Besides, linear peptides are unstructured and have a weak secondary structure in liquor that makes it difficult targeting related receptors [30].

Glycosylation is closely related to protein stability. It can make polypeptide adopt a certain conformation or stabilize the current conformation to improve the hydrophilicity and bioavailability [31–34]. Our team has previously designed an osteoprotegerin- (OPG-) based glycopeptide which is modified with acetylglucosamine. This glycopeptide exhibits a higher secondary structure stability, a stronger affinity to RANKL, and a better resistance to protease cleavage than OPG. Glycosylation renders OPG stronger inhibitory effects on osteoclast formation [35].

In this study, for the first time, our group synthesized a glycosylated teriparatide named PTHG2, and we found that PTHG2 had a better resistance to proteolytic cleavage. Animal experiments showed that PTHG2 preserved bone mass and reduced bone marrow adipocytes. Moreover, we further demonstrated that PTHG2 increased osteogenic differentiation and inhibited adipogenic differentiation of BMSCs through cAMP-PKA signaling pathway. The data of this research suggest that glycosylation is a new modification to improve the clinical utility of PTH (1-34), and PTHG2 could be a potential bone-forming stimulating drug.

## 2. Materials and Methods

### 2.1. Media and Reagents

PTHG2 was kindly provided by Prof. Honggang Hu from Institute of Translational Medicine, Shanghai University. This peptide was generated by a general program of Fmoc solid-phase peptide synthesis (purity > 99%). PTH (1-34) was provided from Beyotime Biotechnology Co. Ltd. (Shanghai, China, purity > 99%) and dissolved in ultrapure water. C57BL/6 mouse bone marrow mesenchymal stem cells complete medium (MUBMX-90011), osteogenic induction medium (MUBMX-90021), and adipogenesis induction medium (MUBMX-90031) were purchased from Cyagen Biosciences (Soochow, China). We bought 5-bromo-4-chloro-3-indolyl phosphate (BCIP)/Nitro Blue Tetrazolium (NBT) Alkaline Phosphatase Color Development Kit (C3206) from Beyotime Biotechnology. In addition, we purchased Alizarin Red S Solution (1%, pH 4.2; G1452) and Oil Red O stain kit (For Cultured Cells) (G1262) from Solarbio Life Sciences (Beijing, China).

### 2.2. Real-Time Quantitative PCR

We got Prime Script RT Master Mix (RR036A) and SYBR Premix Ex Taq (RR420A) from Takara Bio Inc. (Shiga Prefecture, Japan). The total RNA 1 mg extracted from Prime Script RT Master Mix and TRIzol was used to reverse transcription for complementary DNA (cDNA) and as a model for latter real-time qPCR reaction. QTOWER real-time PCR Thermal Cycle Analyzer was used for the QPCR process. The reaction mixture contained forward and reverse primers, SYBR Green Premix Ex Taq and cDNA. The reaction condition was 95°C for 3 minutes, following cycle 40 times at 95°C for 10 seconds, 60°C for 20 seconds, 72°C for 20 seconds, and final 20 seconds at 72 degrees. The primers used in this paper are as follows: *GAPDH* (Forward: 5′-ACCCAGAAGACTGTGGATGG-3′, Reverse: 5′-CACATTGGGGGTAGGAACAC-3′); BGLAP (Forward: 5′-GGTGCAGACCTAGCAGACACCA-3′; Reverse: 5′-AGGTAGCGCCGGAGTCTATTCA-3′); *Osterix* (Forward: 5′-CTTCCCAATCCTATTTGCCGTTT-3′, Reverse: 5′-CGGCCAGGTTACTAACACCAATCT-3′); *Runx2* (Forward: 5′-CCATAACGGTCTTCACAAATCCT-3′; Reverse: 5′-TCTGTCTGTGCCTTCTTGGTTC-3′); *Fabp4* (Forward: 5′-GTAAATGGGGATTTGGTCAC; Reverse: TATGATGCTCTTCACCTTCC); *PPARγ* (Forward: 5′-GCGATTCCTTCACTGATAC-3′; Reverse: 5′-GCATTATGAGCATCCCCAC-3′); *Adipo* (5′-TGTTCCTCTTAATCCTGCCCA-3′ and 5′-CCAACCTGCACAAGTTCCCTT-3′). Data were standardized to GAPDH using 2^−△△CT^ method.

### 2.3. Immunofluorescence and Histological Assessment

Primary antibodies: anti-Osteocalcin antibody (ab93876, 1 : 250), anti-GAPDH antibody-loading control (ab9485, 1 : 10000), and recombinant anti-FABP4 antibody (EPR3579 (ab92501, 1 : 1000) were obtained from Abcam (Cambridge, UK). Runx2 (D1L7F) rabbit mAb (12556, 1 : 1000), PPAR*γ* (C26H12) rabbit mAb (2435, 1 : 1000), adiponectin (C45B10) rabbit mAb (2789, 1 : 1000), CREB (48H2) rabbit mAb (9197, 1 : 1000), and phospho-CREB (Ser133) (87G3) rabbit mAb (9198, 1 : 1000) were purchased from Cell Signaling Technology (Danvers, MA), fluorescently labeled secondary goat anti-rabbit IgG antibody (Dy Light), goat anti-rabbit IgG H&L (HRP) (ab6721, 1 : 1000), and goat anti-rabbit IgG (Alexa Fluor® 488) (ab150077, 1:100) were obtained from Abcam (Cambridge, UK). Primer pairs were purchased from Sangon Co. (Shanghai, China). Recombinant parathyroid receptor 1 (PTH (1-34) R1) protein was purchased from Lianmai Bio-Engineering Co. Ltd. (Shanghai, China). H89-2HCl (S1582) was purchased from Selleck chemicals (Texas, United States). The purity of other chemicals was highest available. For immunofluorescence staining, the femur was decalcified 21 days and embedded in paraffin. The section thickness was 4 *μ*m. After dewaxing and hydrogen peroxide treatment, the antigen repair process was carried out at 65°C for 30 min and 93°C for 13 min using sodium citrate buffer. The samples were sealed with 10% goat serum at normal temperature for 1 h and reacted with primary antibody at 4°C for 14-16 hours. After washing, the samples were reacted with goat anti-rabbit IgG (Alexa Fluor® 488) at room temperature for 1 h, followed by staining with DAPI. For immunohistochemical experiments, the same procedures were used for section process. After overnight incubation, the goat anti-rabbit IgG (HRP) was incubated with goat anti-rabbit at room temperature for 1 h for subsequent DAB reaction. For H&E staining, slices were dewaxed, dyed with hematoxylin and eosin, and finally, processed with hydrochloric acid and ethanol.

### 2.4. Protease Stability Experiment

The purified PTH (1-34) and PTHG2 were dissolved in DMSO at concentration of 1 mg/ml as liquor A. Dissolve *α*-chymotrypsin in CaCl_2_ and PBS liquid (pH = 7.4, including 2 mM CaCl_2_) as solution B to an ultimate concentration of 0.15 ng/*μ*l. 5 *μ*l of liquor A was mixed with 195 *μ*l of solution B. After incubation in 37° for 0, 10, 20, 30, 40, 60, 90, 120, and 180 minutes, high-performance liquid chromatography method was used to detect the peptide residues.

### 2.5. CD Spectroscopy Study

CD parameters was collected on a JASCO J-820 spectropolarimeter (JASCO Corp., Ltd). PTH (1-34) and PTHG2 polypeptides were dispersed in 50.0% TFE solution (TEF : pure aqueous = 1 : 1) to an eventual concentration of 50 *μ*M. The sample ellipticity data were collected at 25°C and 195 nm. The helicity of the polypeptide is calculated by the following formula [36, 37].

(1) $$\begin{array}{c} \alpha =\frac{{\left[\theta \right]}_{222}}{\;{\left[\theta \right]}_{\max}}\times 100\%. \end{array}$$

[*θ*]_222_ was the molar ellipticity at the absorption wavelength of 222 nm, [*θ*]_max_ = (−4400 + 250 T) (1 − *K*/*N*), *K* = 4, and *N* was the number of amino acids of the polypeptide (*n* = 34).

### 2.6. Surface Plasmon Resonance (SPR)

The SPR test was carried out using a Biacore T200 system (GE Healthcare, Sweden) with a CM5 chip at 25°C. PBS was used to dissolve PTH (1-34) R1 on CM5 chip, and the optimal concentration was 50 *μ*g/ml. SPR was used to detect the affinity of PTH (1-34) and PTHG2 solutions, and the contact time was 60 s. Then, different concentrations of PTH (1-34) and PTHG2 were appended at the same flow rate and contact time for kinetic analysis.

### 2.7. OVX-Induced Bone Loss Model

Seventy-two female C57/BL6 mice of 11 weeks old were distributed into six groups (*n* = 12 per group): sham (sham surgery with saline solution), OVX group (with saline solution injection), OVX with low-dose PTH (1-34) (20 *μ*g/kg), OVX with high-dose PTH (1-34) (40 *μ*g/kg), OVX with low-dose PTHG2 (20 *μ*g/kg), and OVX with low-dose PTHG2 (20 *μ*g/kg). After intraperitoneal injection of pentobarbital (40 mg/kg) anesthesia, all mice except sham group proceed bilateral ovariectomy to cut off ovaries and ligated oviduct. One week after surgery, mice were given intraperitoneal injection of corresponding medication daily for 8 weeks. After 8 weeks of therapy, the mice were sacrificed and immobilized with 4% PFA for 48 h. Micro-CT scan and histological evaluation were performed.

### 2.8. Microcomputed Tomography (Micro-CT)

Three-dimensional reconstruction of the collected femur was performed using a SkyScan1275 high-resolution ratio micro-CT scanner (Bruker, Billerica, USA). For the femur sample, the voltage and current of the image acquisition parameters were 50 kV and 60 *μ*A, and the isotropic resolution was 11.5 *μ*m. Trabecular thickness (Tb. Th, mm), ratio of bone surface area to bone volume (BS/BV, mm), bone volume fraction (BV/TV, %), trabecular number (Tb. N, mm), trabecular spacing (Tb. Sp, mm), and ratio of bone surface area to tissue volume (BS/TV, mm) were calculated.

### 2.9. BMSC Isolation

BMSCs were extracted from the femoral and tibial marrow cavity of 5-week-old C57/BL6 mice and cultured in mouse bone marrow mesenchymal stem cell medium for 2-3 days. The new culture medium was changed until the adherent cells grew 95% confluence and cell passage, or subsequent experiments were carried out. The cell growth environment was 37°C, 5% CO_2_, and 10% humidity.

### 2.10. Cell Viability Assay

Cell viability of BMSCs were tested using the CCK-8 (Dojindo Molecular Technology, Kumamoto, Japan) according to instruction. BMSCs were inoculated in a 96-well plate (5 × 10^3^ cells per well) and hatched overnight. On the second day, cells were treated with PTH (1-34)/PTHG2 with different concentrations (0, 0.005, 0.01, 0.1, 1, and 100 *μ*M) for 48 h and 96 h. Then, cells were disposed with 10 *μ*l of CCK-8 reagent for 2 hours. After incubation, the absorbance was measured at 450 nm in a Biotek Cytation 5 System (Biotek Instruments, Vermont, USA).

### 2.11. In Vitro Osteogenesis Assay

BMSCs were cultured in 12-well plates (5 × 10^4^ per well). When the density of cell reached 90%, the basic medium was exchanged with osteogenic differentiation medium of mouse BMSCs, and the medium was replaced every two days. After the induction of 7 days, the cells were immobilized with 4% PFA for 15 min, and the osteogenic differentiation ability was tested using the BCIP/NBT Color Development Kit. In order to test the ability of bone mineralization, BMSCs were treated according to the above method for 21 days. After treatment, the cells were immobilized with 4% PFA for 15 min, washed with 1× PBS for three times, and then saturated with 1% Alizarin Red S solution at room temperature for 30 minutes. After the plate was air-dried, the image was carried out with a microplate analyzer, and the ALP and mineralization degree were measured with Image J (Edition, Manufacturer).

### 2.12. In Vitro Adipogenesis Assay

BMSCs were inoculated into 12-well plates (2 × 10^4^ per well). When the degree of cell confluence reached 100%, liquid A of mouse BMSC cell adipogenesis induction differentiation medium was added into the wells. After 3 days of induction, liquid B replaced A for 1 day. The induction and maintenance process were repeated until sufficient lipid droplets of appropriate size are present. The oil red O staining kit was used, and the oil red staining was executed according to manufacturer's instructions.

### 2.13. Western Blotting

To investigate whether PTHG2 worked through the cAMP-PKA signaling pathway, BMSCs were first starved for 1 hour, and cells were treated with or without H89-2HCl for 1 hour and then stimulated with 10 *μ*M PTH (1-34)/PTHG2 for 6 hours, respectively. The unstimulated group was the control group. After the stimulation, the cells were digested, and RIPA cell lysate (containing protein phosphatase inhibitors) was added and centrifuged. 30 *μ*g protein was separated with SDS-PAGE. The proteins were then shifted to the nitrocellulose membrane using a trans-Blot Turbo Transfer System (Bio-Rad Laboratories, Hercules, CA, USA). Membrane was sealed with 5% bovine serum albumin (BSA) for 1 h. Membrane and specific primary antibody were incubated overnight at 4°C. Then, membrane was disposed with fluorescently labeled secondary Goat anti-Rabbit IgG Antibody (Dylight) for 1 hour at 25°C. The protein bands were imaged with a Bio-Rad visual imaging system (Bio-Rad Laboratories, Hercules, CA), and the relative protein expression was analyzed by Image J.

### 2.14. Animal Ethics Statement

All animal models were conducted on the basis of the approval of the Ethics Committee of Shanghai University. All the animals were kept in a feeding environment with a 12-hour day/night cycle at 22-25°C.

### 2.15. Statistical Analyses

All data were analyzed by GraphPad Prism8 software and presented as means plus standard deviation. Independent *t*-test was used to examine the discrepancies between the two groups. Meanwhile, one-way ANOVA was used for statistical analysis across multiple samples. Each *in vitro* experiment was repeated three times. *p* value less than 0.05 represented a statistically significance.

### 2.16. BrdU Staining

Cells were cultured with 10 *μ*M BrdU for one hour and fixed in 70% ethanol at -20 C. After washing with phosphate/citric buffer (40 ml Na_2_HPO_4_ with 4 ml 0.1 M citric acid), the cells were incubated with anti-BrdU antibody for one hour, then stained with propidium iodide (PI). For PI/RNase staining, cells were incubated with 0.5 ml of PI/RNAse staining buffer (BD Pharmingen) at 25°C for 15 minutes, then analyzed by flow cytometry.

## 3. Results

### 3.1. PTHG2 Shows Better Pharmacological Properties Than PTH (1-34) In Vitro

**Figure 1:**
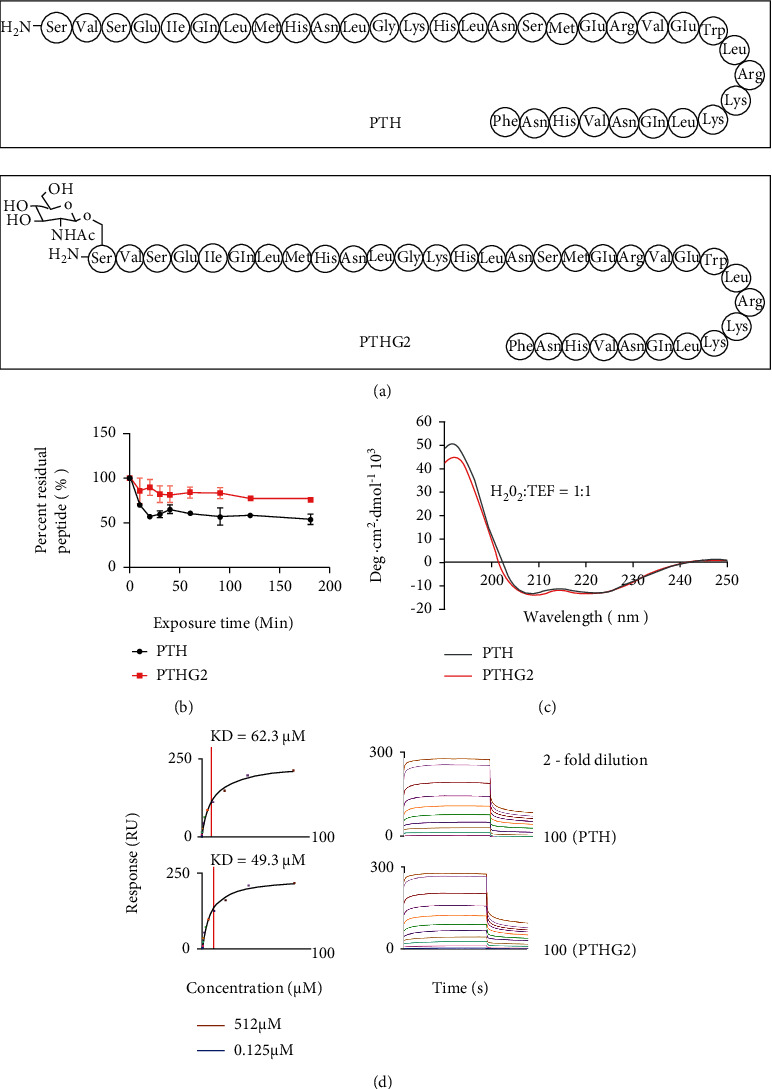
The stability of PTHG2 is better than that of PTH (1-34). (a) The structural formula of PTHG2; (b) the resistance of PTH (1-34) and PTHG2 to proteolytic cleavage was measured; (c) CD spectra of PTHG2 in 50.0% TFE water solution at 20°C; (d) the affinity between PTHG2 and PTHR1. Data appeared as mean ± standard deviation (*n* = 3).

PTHG2 was a glycosylated derivative obtained by acetylglucosamine modification of PTH (1-34) N-terminal serine (Figure 1(a)). PTH (1-34) and PTHG2 were treated with chymotrypsin, and the antiproteolytic stability was detected. Results showed that the degradation of PTH (1-34) polypeptide tended to stabilize at 55%, and PTHG2 was maintained at 83% (Figure 1(b)) after 90 min at the same concentration. The secondary structure of the synthetic glycopeptide was analyzed by CD. The helicity of PTH (1-34) was 38.5% lower than 39.1% of PTHG2 (Figure 1(c)). To test whether PTHG2 activated PTHR1, we ran the affinity detection module to conduct SPR affinity test, and the KD values of PTH (1-34) and PTHG2 were 62.3 *μ*M and 49.3 *μ*M (Figure 1(d)). Taken together, PTHG2 had better protease stability, peptide helicity value, and analogous PTHR1 affinity compared with PTH (1-34).

### 3.2. Intermittent Administration of PTHG2 Alleviates Bone Loss in OVX Mice Model

**Figure 2:**
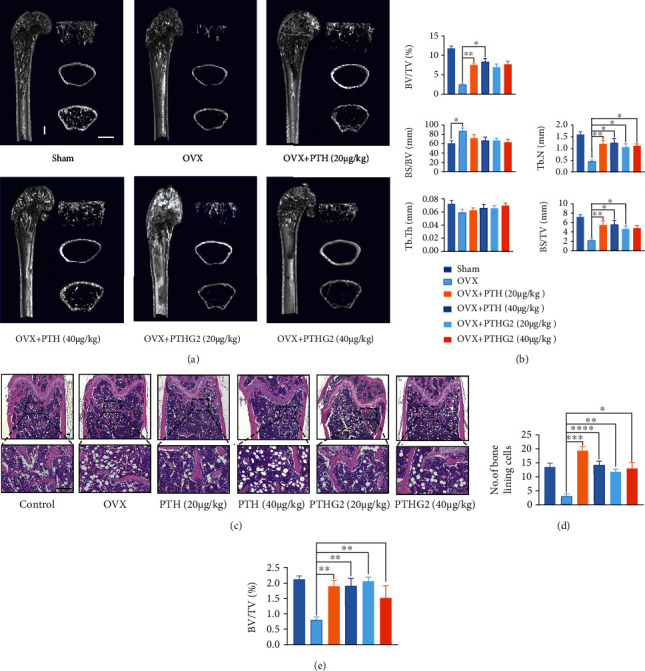
Intermittent injection of PTHG2 alleviated bone loss in mice induced by OVX. (a) Representative micro-CT images of femora of control group and curative groups; scale bar, 1 mm; (b) quantitative micro-CT of bone volume per tissue volume (BV/TV), number of trabeculae (Tb. N), relative bone volume fraction (BS/TV), bone surface area/bone volume (BS/BV), trabecular thickness (Tb. Th), and bone surface area/tissue volume (BS/TV); (c) H&E staining of femora. Scale bar, 100 *μ*m; (d) quantitative number of lining cells. (e) Quantitative H&E staining of bone volume per tissue volume (BS/TV). Scale bar, 30 *μ*m, *n* = 6 per group (^∗^*p* < 0.05, ^∗∗^*p* < 0.01, ^∗∗∗^*p* < 0.001, ^∗∗∗∗^*p* < 0.001).

In order to test the protective effect of PTHG2 on bone loss *in vivo*, we performed OVX surgery to simulate postmenopausal bone loss. Micro-CT results exhibited that bone volume fraction (BV/TV) and bone trabeculae number (Tb. N) were significantly decreased after OVX operation compared with sham group. In comparison to OVX group, BV/TV, Tb. N, and BS/TV (relative bone volume fraction) significantly increased after intermittent disposal of PTH (1-34) (20/40 *μ*g·kg^−1^). Similarly, intermittent usage of PTHG2 (20/40 *μ*g·kg^−1^) also had a similar effect on bone mass (Figures 2(a) and 2(b)). Histological evaluation showed that the number of lining cells on the trabecular bone surface after PTHG2 treatment was significantly increased compared to the OVX group (Figures 2(c) and 2(d)). The bone volume fraction of mice was also similar to that of the PTH (1-34) group, indicating that intermittent administration of PTHG2 could achieve the comparable bone mass recovery effect as that of PTH (1-34) (Figure 2(e)).

### 3.3. PTHG2 Protects the Reduced Osteoblast Activity Induced by OVX and Weakens the Formation of Bone Marrow Adipocytes

**Figure 3:**
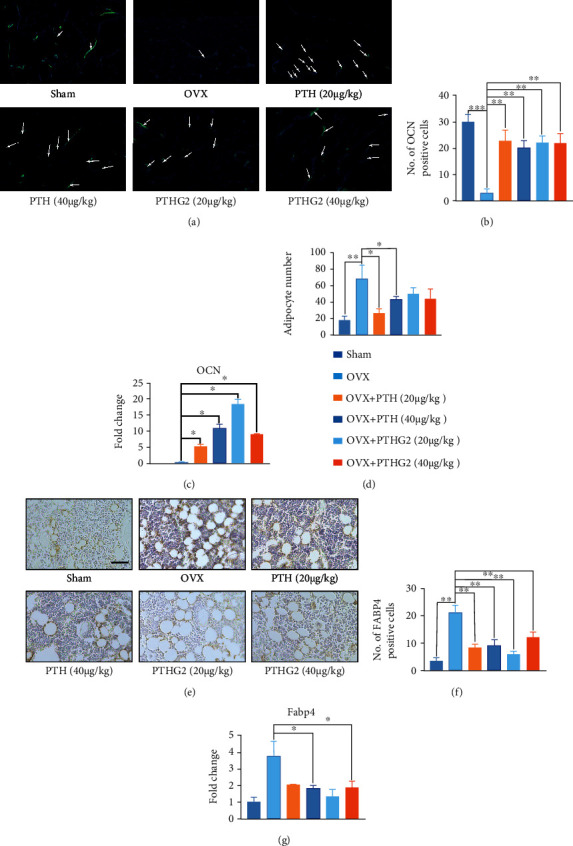
PTHG2 protects the reduced osteoblast activity induced by OVX and weakens the formation of bone marrow adipocytes. (a) Representative images for immunofluorescence assay of osteocalcin (OCN). (b) Quantitative positive cells of OCN. (c) The relative expression of osteoblast marker gene (OCN) following PTHG2 treatment was quantified by real-time PCR. (d) Quantitative lipid droplets of H&E staining. (e) Representative images for immunohistochemistry assay of Fabp4 and (f) quantity. (g) The relative expression of adipocyte marker gene (Fabp4) following PTHG2 treatment was quantified by real-time PCR. Values presented as the mean ± standard deviation (*n* = 3); scale bar, 50 *μ*m; ^∗^*p* < 0.05, ^∗∗^*p* < 0.01, ^∗∗∗^*p* < 0.001, ^∗∗∗∗^*p* < 0.001.

To further illustrate the potential mechanisms of PTHG2-mediated bone protection, we performed a histological evaluation. Histological images of OCN staining of the distal femur displayed a significant increase in OCN positivity in the PTHG2-treated group (Figures 3(a) and 3(b)), indicating that PTHG2 increased the level of BMSCs that differentiated into osteoblasts. At the same time, we extracted the bone mRNA of the upper limb of mice for qPCR analysis. Compared with the OVX group, the expression of OCN gene was significantly increased after PTHG2 treatment (Figure 3(c)). The adipocytes in HE staining images were analyzed by counting vacuoles (Figure 3(d)). The results showed that the adipocytes in the metaphysis of OVX group significantly increased. Compared with OVX group, the number of cavitation was significantly reduced after PTHG2 treatment. To further evaluate the adipogenesis, we performed histological evaluation of FABP4 protein in adipocytes of the distal femur by IHC (Figure 3(e)). As exhibited in staining image (Figure 3(f)), the number of FABP4-positive cells in the OVX group was increased compared with the control group, and PTHG2 reduced the number of FABP4-positive cells in OVX mice. PTHG2 impaired OVX-induced adipocyte formation. We also confirmed the conclusion at the gene level. QPCR results confirmed that PTHG2 could achieve the same effect as PTH (1-34) in inhibiting adipocyte generation (Figure 3(g)). All the above results suggested that the positive effect of PTHG2 on bone mass was caused by the regulation of adipo-osteogenic differentiation in BMSCs.

### 3.4. Intermittent PTHG2 Accelerates Osteogenic Differentiation of BMSCs and Restrains Adipogenic Differentiation In Vitro

**Figure 4:**
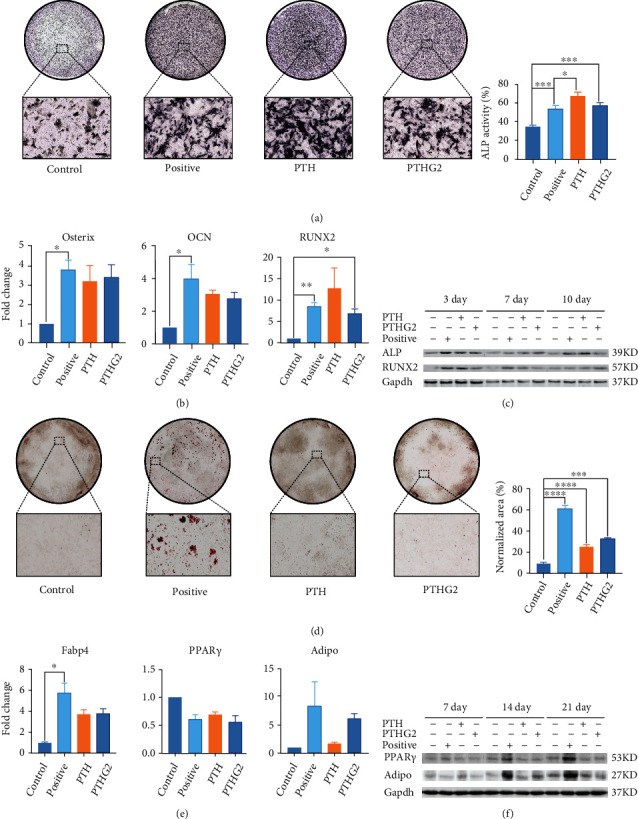
PTHG2 promotes osteogenic differentiation of BMSCs and inhibits adipogenic differentiation. (a) BMSCs were cultured in osteoblast medium for 7 days, and the ALP positive area was measured. (b) The relative expression of osteoblast marker gene (OCN/Osterix/RUNX2) following PTHG2 treatment was quantified by real-time PCR. (c) Western blotting assays of ALP and RUNX2 during osteogenic differentiation. (d) Oil red O staining detected the effects of PTHG2 on BMSCs adipogenic differentiation and quantity. (e) The relative expression of adipocyte marker gene (Fabp4/PPARɣ/Adiponectin) following PTHG2 treatment was quantified by real-time PCR. (f) Western blotting assays of PPARɣ and adiponectin during adipogenic differentiation. Scale bar = 30 *μ*m (*n* = 3); ^∗^*p* < 0.05, ^∗∗^*p* < 0.01, ^∗∗∗^*p* < 0.001, ^∗∗∗∗^*p* < 0.001.

To further verify that PTHG2 interfered adipo-osteogenic differentiation balance of BMSCs, we conducted a series of *in vitro* experiments. Before evaluating the effect of PTHG2 on osteogenic differentiation and adipogenic differentiation of BMSCs, CCK-8 activity assay was carried out. Results showed that PTHG2 had no toxicity to BMSCs at concentrations below 100 *μ*M (Supplementary Materials 1(a)). In addition, we conducted in vivo tissue toxicity test and found that PTHG2 had no toxicity to the heart, liver, spleen, lung, and kidney of mice (Supplementary Materials 2(a)). PI staining was performed on the cells, and cell cycle was analyzed by flow cytometry. It was found that PTHG2 had no effect on cell cycle (Supplementary Materials 1(b)). The effect of PTHG2 on osteogenic differentiation and mineralization of BMSCs were examined. BMSCs were stimulated with 10 nM PTH (1-34) and PTHG2 in the presence of *β*-sodium ascorbic acid and glycerophosphate and dexamethasone in conditioned medium for 7 and 21 days (6 h/day), respectively. After 7 days of differentiation, the PTHG2-treated group significantly enhanced the osteogenic differentiation activity of BMSCs (Figure 4(a)) and bone mineralization (Supplemental Materials 1(c)). Meanwhile, RNA from cell samples was extracted, and the gene expression of Osterix, OCN, and Runx2 in the differentiation process was detected. The expression of Runx2 in PTHG2-treated group was higher than that in control group (Figure 4(b)). Protein levels was also examined at 3, 7, and 10 days, and results showed that PTHG2 promoted osteogenic differentiation of BMSCs in a time-dependent manner. Besides, ALP and RUNX2 protein levels were significantly increased compared to the control group (Figure 4(c)). Then, we examined the effect of PTHG2 on the adipocyte differentiation of BMSCs. BMSCs were induced into adipogenesis in conditioned medium containing insulin, rosiglitazone, IBMX, and dexamethasone for 21 days and then infiltrated with. The consequences of the oil red staining showed that, compared with the control, PTHG2 did not increase the number of adipocytes (Figure 4(d)). We also tested the gene and protein levels which showed that the gene protein expression of the PTHG2-treated samples was resemble to that of the control group (Figures 4(e) and 4(f) and Supplementary Materials 1(d)). The above results confirmed that intermittent administration of PTHG2 *in vitro* changed the balance of adipo-osteogenic differentiation of BMSCs, promoted the differentiation of BMSCs into osteoblasts, and inhibited the differentiation of BMSCs into adipocytes.

### 3.5. PTHG2 Enhances the Differentiation of BMSCs into Osteoblasts by Stimulating cAMP-PKA Pathway

**Figure 5:**
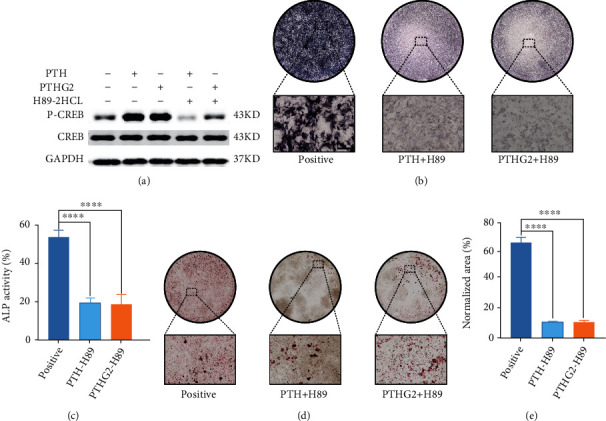
PTHG2 stimulates osteogenic differentiation of BMSCs through cAMP-PKA signaling. (a) Densitometry analysis of P-CREB protein levels. (b) ALP staining and (c) quantification after H89-2HCL pretreatment. (d) Oil red O staining and (e) quantity after H89-2HCL pretreatment. Scale bar, 30 *μ*m (*n* = 3); ^∗^*p* < 0.05, ^∗∗^*p* < 0.01, ^∗∗∗^*p* < 0.001, ^∗∗∗∗^*p* < 0.001.

**Figure 6:**
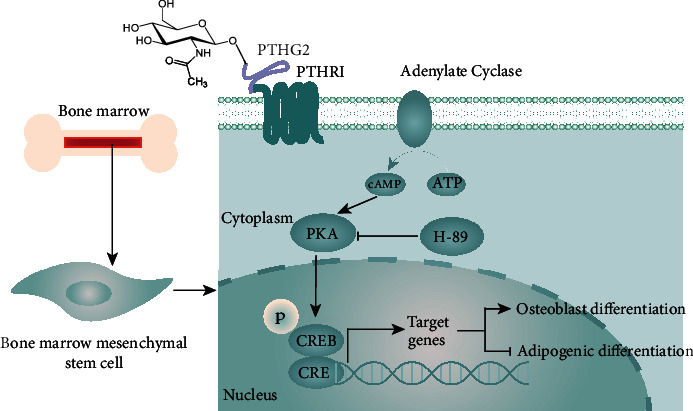
PTHG2 interacts with PTH (1-34) R1 to activate adenylate cyclase (AC), activate second messenger cAMP-dependent protein kinase (PKA), phosphorylation CREB into nucleus, and control downstream target genes expression, such as osteogenic genes (OCN/RUNX2/Osterix) and adipocyte production markers (Fabp4/PPARɣ/Adiponectin).

cAMP-PKA signaling pathway was a crucial mechanism of PTH (1-34) stimulating bone anabolism [38, 39]. After PTH (1-34) activated PTHR1 on the surface of cell membrane, classical G protein signaling cascade occurred. Activated adenylate cyclase (AC) signaling cascade leaded to excitation of protein kinase A (PKA) and phosphorylation of transcription factor CREB. P-CREB regulated PTH (1-34) downstream genes [40]. H89-2HCl was a PKA inhibitor, which was used to study the activation of cAMP-PKA signaling pathway. The above SPR experiment confirmed that PTHG2 also activated PTHR1 receptor. To verify whether PTHG2 phosphorylated CREB through the cAMP-PKA signaling pathway, CREB transcription factor protein levels were first measured. BMSCs were stimulated with PTH (1-34) and PTHG2 for 6 hours with or without the addition of H89-2HCl. Cell proteins were extracted, and the protein expressions of CREB and p-CREB were detected (Figure 5(a)). The consequences showed that PTHG2 increased the phosphorylation level of CREB. The phosphorylation level of CREB was not increased in H89-2HCL group, which confirmed that PTHG2 stimulated the phosphorylation of CREB. Then, we used H89-2HCl to interfere with the process of osteogenic differentiation and mineralization of BMSCs. The results affirmed that PTHG2 stimulated osteogenic differentiation of BMSCs through the cAMP-PKA signaling pathway (Figures 5(b) and 5(c)). Interestingly, the adipogenic differentiation activity of BMSCs was enhanced after PKA signal blocking (Figures 5(d) and 5(e) and Supplementary Materials 1(e)). In general, the N-terminal acetylglucosamine-modified derivative of PTH (1-34) activated the cell membrane surface receptor PTHR1 of BMSCs, activated AC, stimulated PKA, and phosphorylated CREB transcription factor, thus activating osteogenic-related target genes and inhibiting adipogenesis-related target genes (Figure 6).

## 4. Discussion

Osteoporosis is a serious disease in the aged population which currently affects 200 million people worldwide and causes approximately 8.9 million fractures every year [41, 42]. About 21.4% of postmenopausal women over the age of 50 may have osteoporosis, and 51.2% of them have significant bone mass loss [43]. Though the needs of treating osteoporosis are urgent, well-recognized therapeutic methods are still absent.

Developing antiosteoporosis drugs based on a fundamental conception that osteoporosis is a metabolic bone disease caused by overactivation of bone resorption and low activity of bone formation. This imbalance decreases bone mass, destroys microstructure of bone, and increases bone fragility which leads to high risk of fragile fracture. Comparing with many antibone resorption drugs such as bisphosphonates, RANK ligand (RANKL) inhibitor, and selective estrogen-receptor modulator (SERM), the number of bone formation stimulating agents is less because of unclear regulatory mechanism of BMSC osteogenic differentiation. BMSCs are a group of newly discovered mesenchymal stem cells in recent years [44]. BMSCs are self-renewal, three-line differentiation (osteogenesis, adipogenesis, and chondrogenesis) and show strong immune regulation role [45]. Most of osteoblasts and adipocytes in bone marrow are derived from BMSCs [8].

The unbalanced differentiation of BMSCs is one of the causes of osteoporosis [46]. Latest researches have shown that osteoporosis is accompanied by a boost of bone marrow adipose tissue [47]. Another research has confirmed that the number of osteoblasts decreases and adipocytes increases in the marrow of patients with osteoporosis [48]. BMSCs taken from clinical samples of women with osteoporosis and the control group are compared, and differences are found in the ability of osteogenic and adipogenic differentiation [49]. The decreased ability of BMSCs to produce osteoblasts makes them more prone to produce adipocytes, leading to osteoporosis and even fragility fractures [50].

Regulating the differentiation fate of BMSCs is a potential treatment option for osteoporosis. Several studies have revealed that PTH (1-34) could promote BMSC osteogenic differentiation and inhibit adipocyte formation [51, 52]. PTH (1-34) has been revealed to enhance bone formation, and intermittent low-dose application of PTH (1-34) can increase osteoblast differentiation and suppress adipocyte differentiation of BMSCs [53]. In the OVX model of rats, osteogenic gene expression is increased, and adipogenesis gene expression is decreased after PTH (1-34) treatment, such as PPARɣ. PTH (1-34) promotes BMSC osteogenic differentiation and inhibits adipocyte formation [51, 52]. PTH (1-34) stimulates bone anabolism by activating the intracellular second messenger cAMP [54]. PKA, as a downstream signaling pathway of cAMP, is stimulated by PTH (1-34) to phosphorylate CREB and activate downstream osteogenic differentiation marker genes [54–56].

Protein glycosylation can improve stability of PTH (1-34). PTH (1-34) is the sole synthetic drug applied to the treatment of postmenopausal osteoporosis, glucocorticoid-related osteoporosis, and osteoporosis with high risk of fracture [57]. However, as a long chain linear polypeptide, PTH (1-34) has disadvantages such as short action time and unstable secondary structure in solution. Using appropriate modification to offset those disadvantages may improve the willingness of using PTH (1-34). Protein glycosylation is an important posttranslation modification [31]. Glycosylation increases peptide bioavailability and hydrophilicity, induces peptides to employ certain conformations and/or stabilizes the conformations in existence, and heightens peptide resistance to proteolytic cleavage. Levine finds that O-Glycosylation of glucagon-like peptide 1 (GLP-1) and PTH results in higher stability in serum and increased *in vivo* activity, suggesting that O-Glycosylation enhances the stability of proteolysis [58]. Ueda et al. perform N-acetylglucosamine acylation of glucagon-like peptide 1 (7-36) amide (GLP-1) to improve the stability [59]. Federica performs monosaccharide of GC-MAF, and the results show that the glycosylation has good tolerance to the secondary and tertiary structures of the polypeptide scaffold [60].

In this study, our team synthetized a modified PTH (1-34) called PTHG2 which was added an acetylglucosamine to the first serine at N-terminal. We further confirmed that glycosylation of PTH (1-34) did not change the binding ability of PTHR1 according to SRP affinity assay. PTHR1 was a kind of receptors that existed on the cell membrane and binded to PTH (1-34). The antichymotrypsin stability assays showed that stability of PTH (1-34) was improved after glycosylation, which may be caused by more helical secondary conformations in solution. The animal experiments by using micro-CT and HE staining confirmed a favourable antiosteoporosis protective effect of glycosylated PTH (1-34). By using some *in vitro* experiments, we found that PTHG2 could promote BMSC osteogenic differentiation and inhibit BMSC adipogenic differentiation. This suggested that PTHG2 changed the fate of BMSC differentiation and promoted bone formation, thereby breaking the metabolic balance between osteoblasts and osteoclasts and ultimately increasing bone mass. We also found that PTHG2 activated osteogenic genes by exciting the cAMP-PKA signaling pathway.

## 5. Conclusions

In this study, we found that a glycosylation-modified PTH (1-34) named PTHG2 exhibited higher stability and better antiosteoporosis effect than PTH (1-34). PTHG2 accelerated the differentiation of BMSCs to osteogenic lineage and restrained the differentiation of BMSCs to adipogenic lineage via cAMP/PKA/CREB signaling pathway. Therefore, PTHG2 could be a potential therapeutic drug for osteoporosis as a reformed PTH (1-34).

## Data Availability

The original contributions included in this study are contained in the article and supplementary materials, and further suggestions can be made to the corresponding author or first author.

## Supplementary Materials

Supplementary Materials Supplementary Figure S1 PTHG2 did not affect the proliferation of BMSCs and promoted the osteogenic differentiation of BMSCs and inhibited the differentiation into adipocytes. (a) BMSC cell viability as assessed by CCK-8 assay following treatment without or with indicated concentrations of PTHG2 for 48 and 96 h. (b) Alizarin red staining of osteogenic differentiation with PTH (1-34) or PTHG2 and quantify. (c) The relative expression of Cebp-*α* and Perilipin following PTHG2 treatment was quantified by real-time PCR. (d) After pretreatment with H89, BMSC differentiated and stained with Alizarin red for 21 days. Supplementary Material 2: H&E staining of the heart, liver, spleen, lung, and kidney after administration of normal saline, PTH, and PTHG2.
