# Supplementary material for: PTHG2 Reduces Bone Loss in Ovariectomized Mice by Directing Bone Marrow Mesenchymal Stem Cell Fate
Source: Stem Cells Int. 2021 Nov 19;2021:8546739. doi: 10.1155/2021/8546739 (PMC8720025; doi:10.1155/2021/8546739)
Supplement: Supplementary Materials — Supplementary Figure S1 PTHG2 did not affect the proliferation of BMSCs and promoted the osteogenic differentiation of BMSCs and inhibited the differentiation into adipocytes. (a) BMSC cell viability as assessed by CCK-8 assay following treatment without or with indicated concentrations of PTHG2 for 48 and 96 h. (b) Alizarin red staining of osteogenic differentiation with PTH (1-34) or PTHG2 and quantify. (c) The relative expression of Cebp-α and Perilipin following PTHG2 treatment was quantified by real-time PCR. (d) After pretreatment with H89, BMSC differentiated and stained with Alizarin red for 21 days. Supplementary Material 2: H&E staining of the heart, liver, spleen, lung, and kidney after administration of normal saline, PTH, and PTHG2. [file 8546739.f1.pdf]

Supplementary Materials1

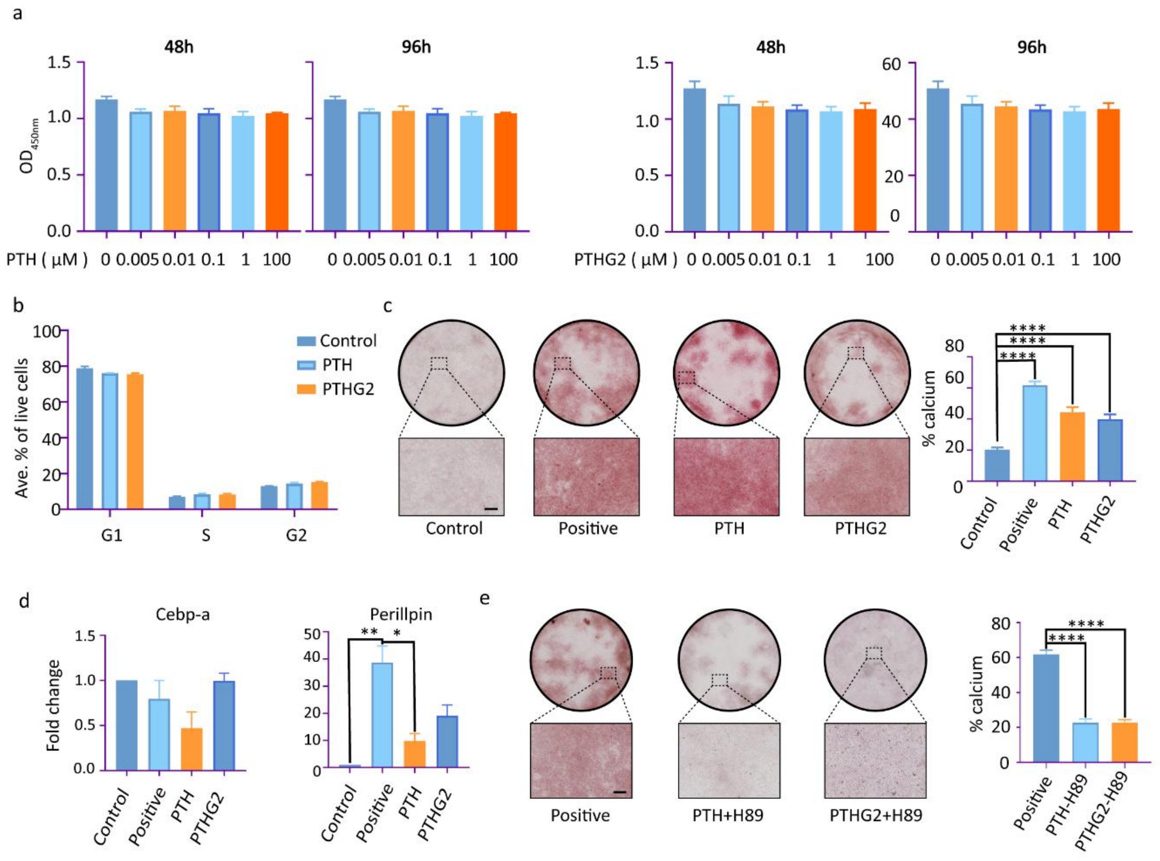

Supplementary material 1: PTHG2 did not affect the proliferation of BMSCs and promoted the osteogenic differentiation of BMSCs and inhibited the differentiation into adipocytes. a) BMSC cell viability as assessed by CCK-8 assay following treatment without or with indicated concentrations of PTHG2 for 48 and 96h. b) The cell cycle is analyzed with PI staining. c) Alizarin red staining of osteogenic differentiation with PTH (1-34) or PTHG2 and quantify. d) The relative expression of Cebp-α and Perilipin following PTHG2 treatment were quantified by real time PCR. e) After pretreatment with H89, BMSC differentiated and stained with Alizarin red for 21 days. Data presented as mean ± standard deviation (n=3). Scale bar, 30µm; *p<0.05, **p<0.01, ***p<0.001, ****p<0.001.

Supplementary Materials2

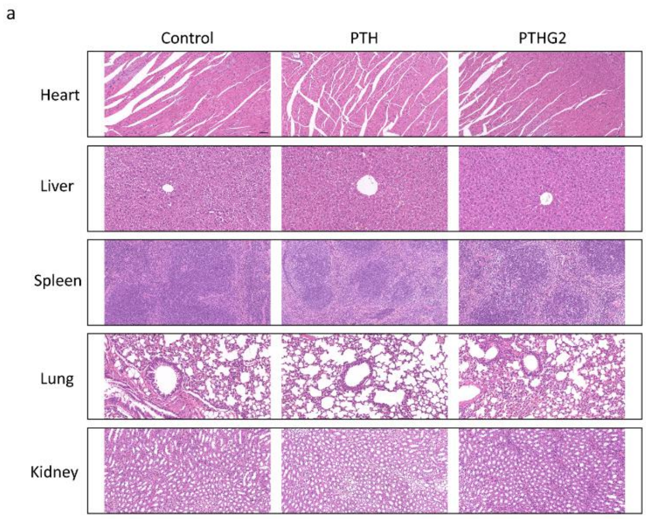

Supplementary material 2: H&E staining of the heart, liver, spleen, lung and kidney after administration of Normal saline, PTH, PTHG2, Scale bar, 50μm.
